# Exponential rise of dynamical complexity in quantum computing through projections

**DOI:** 10.1038/ncomms6173

**Published:** 2014-10-10

**Authors:** Daniel Klaus Burgarth, Paolo Facchi, Vittorio Giovannetti, Hiromichi Nakazato, Saverio Pascazio, Kazuya Yuasa

**Affiliations:** 1 Institute of Mathematics, Physics and Computer Science, Aberystwyth University, Aberystwyth SY23 3BZ, UK; 2 Dipartimento di Fisica and MECENAS, Università di Bari, I-70126 Bari, Italy; 3 INFN, Sezione di Bari, I-70126 Bari, Italy; 4 NEST, Scuola Normale Superiore and Istituto Nanoscienze-CNR, I-56126 Pisa, Italy; 5 Department of Physics, Waseda University, Tokyo 169-8555, Japan

## Abstract

The ability of quantum systems to host exponentially complex dynamics has the potential to revolutionize science and technology. Therefore, much effort has been devoted to developing of protocols for computation, communication and metrology, which exploit this scaling, despite formidable technical difficulties. Here we show that the mere frequent observation of a small part of a quantum system can turn its dynamics from a very simple one into an exponentially complex one, capable of universal quantum computation. After discussing examples, we go on to show that this effect is generally to be expected: almost any quantum dynamics becomes universal once ‘observed’ as outlined above. Conversely, we show that any complex quantum dynamics can be ‘purified’ into a simpler one in larger dimensions. We conclude by demonstrating that even local noise can lead to an exponentially complex dynamics.

## Abstract (web summary)

It is an old adage in quantum physics that the observation of a system changes its properties, as exemplified by the quantum Zeno effect. Now, Burgarth *et al.* show that such repeated measurement of a quantum system actually enriches its dynamics, letting it explore a much larger algebra than it did before.

## Graphical abstract

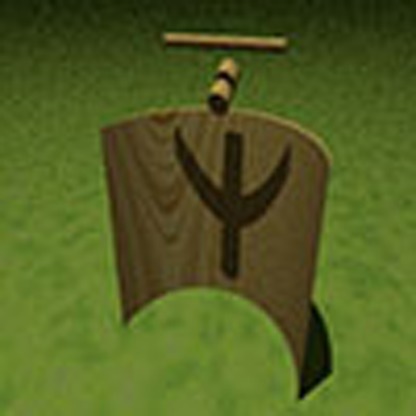

In the last 30 years, the possibility of using quantum effects to develop an alternative approach to engineering has emerged as a realistic way to improve the efficiency of computation, communication and metrology^1^,^2^,^3^,^4^,^5^,^6^. At the very core of this revolutionary idea, the possibility of designing arbitrary dynamics of quantum systems without spoiling the rather fragile correlations characterizing them is crucial. What experimentalists typically do is to apply sequences of control pulses (for example, by sequentially switching on and off different electromagnetic fields) to steer quantum systems. In the quantum world, however, there is another option associated with the fact that the measurement process itself can induce a transformation on a quantum system. In this context, an intriguing possibility is offered by the quantum Zeno effect^7^,^8^. This effect forces the system to evolve in a given subspace of the total Hilbert space by performing frequent projective measurements (Zeno dynamics)^9^,^10^,^11^, without the need of monitoring their outcomes (non-adaptive feedback strategy). Several attempts have already been discussed to exploit such effects for quantum computation, see refs 12, 13, 14, 15, 16, 17, 18, 19, 20, 21, 22.

In this work, we show that the constraint imposed via a Zeno projection can in fact enrich the dynamics induced by a series of control pulses, allowing the system of interest to explore an algebra that is exponentially larger than the original one. In particular, this effect can be used to turn a small set of quantum gates into a universal set. Thanks to the non-adaptive character of the scheme, this Zeno enhancement can also be implemented by a non-cooperative party, for example, by noisy environment. Furthermore, we show that any complex quantum dynamics can be viewed as the projected dynamics of a simpler one in larger dimensions.

By the Zeno effect, the dynamics of the system is forced to evolve in a given subspace of the total Hilbert space^9^,^10^,^11^. One might therefore think that the constrained dynamics is less ‘rich’ than the original one. This naive expectation will turn out to be incorrect. These surprising aspects of constraints bear interesting similarities to Einstein’s precepts, according to which one can give a geometric description of complicated motion. The key geometrical idea is to embed the motion of the system of interest in a larger space, obtaining a forceless dynamics taking place along straight lines. The real dynamics, with interactions and potentials, is then obtained by projecting the system back onto the original space. Clearly, the constrained dynamics is more complex than the higher-dimensional linear one. In classical mechanics, these reduction procedures, linking a given dynamical system with the one constrained on a lower-dimensional manifold, have been extensively studied as an effective method for integrating the dynamics^23^. In particular, different classes of completely integrable systems arise as reductions of free ones with higher degrees of freedom^24^,^25^,^26^. Notable examples include the three-dimensional Kepler problem, the Calogero–Moser model, Toda systems, KdV and other integrable systems. The lesson learned is that in classical mechanics, by constraining the dynamics, one often obtains an increase in complexity.

Here we find a quantum version of this intriguing effect, which exploits the inherent non-commutative nature of quantum mechanics. The main idea is that even if two Hamiltonians *H* and *H*′ are commutative, their projected counterparts can be non-commutative


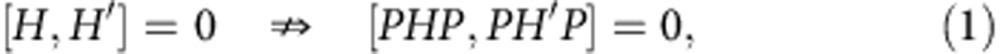


where *P*=*P*^2^ is a projection. Due to this fact, we show that when passing from a set of control Hamiltonians {*H*^(1)^,…, *H*^(*n*)^} to their projected versions {*PH*^(1)^*P*,…, *PH*^(*n*)^*P*} one can induce an enhancement in the complexity of the system dynamics which can be exponential, to the extent that it can be used to transform a small number of quantum gates which are not universal into a universal set capable of performing arbitrary quantum-computational tasks. We find that this effect is completely general and happens in almost all systems. Conversely, we prove that any complex dynamics can be viewed as a simple dynamics in a larger dimension, with the original dynamics realized as a projected dynamics. What is interesting is that, in contrast to the classical case, the constraint which transforms a Hamiltonian *H* into *PHP* can be imposed not by force but by a simple projective measurement whose outcomes need not be recorded (the process being effectively equivalent to the one associated with an external noise that is monitoring the system).

## Results

### Unitary control versus Zeno dynamics

In controlled quantum dynamics, two Hamiltonians can commute, but their projected versions need not. This contains, in embryo, the simple idea discussed in the introductory paragraph: interaction can arise from constraints (in this case projections). To describe this mechanism, it is worth recalling a few facts about quantum control theory and the quantum Zeno effect.

**Figure 1:**
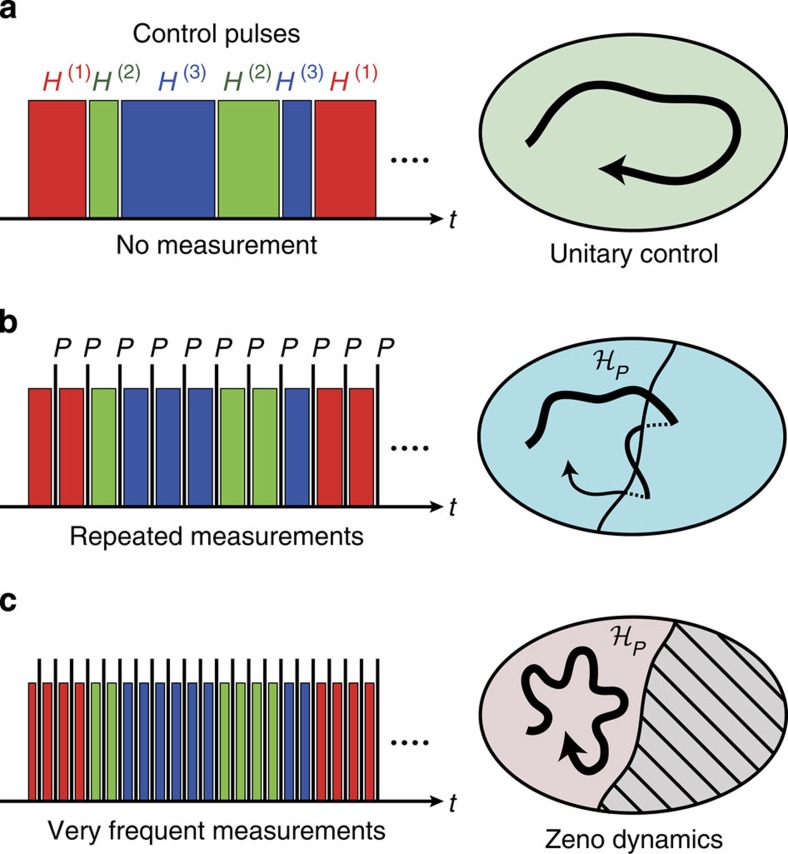
Zeno effect in quantum control. (**a**) We control a quantum system by switching on and off a set of given Hamiltonians {*H*^(1)^,…, *H*^(*n*)^}. (**b**) We perform projective measurements *P* at regular time intervals during the control to check whether or not the state of the system belongs to a given subspace 
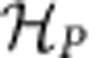
 of the global Hilbert space. (**c**) In the limit of infinitely frequent measurements (Zeno limit), the system is confined in the subspace 
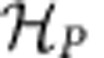
, where it evolves unitarily with the Zeno Hamiltonians {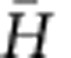
^(1)^,..., 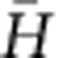
^(*n*)^} (Zeno dynamics). The Zeno dynamics can explore the subspace 
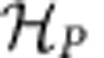
 more thoroughly than the purely unitary control without measurement.

In a typical quantum control scenario, it is assumed that the system of interest (say the quantum register of a quantum computer, or the spins in an NMR experiment) can be externally driven by means of sequences of unitary pulses 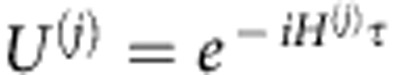, activated by turning on and off a set of given Hamiltonians {*H*^(1)^,…, *H*^(*n*)^} (Fig. 1a). If no limitations are imposed on the temporal durations *τ* of the pulses, it is known^27^ that by properly arranging sequences composed of {*U*^(1)^,…, *U*^(*n*)^} one can in fact force the system to evolve under the action of arbitrary transformations of the form *U*=*e*^Θ^ with the anti-Hermitian operators Θ being elements of the real Lie algebra 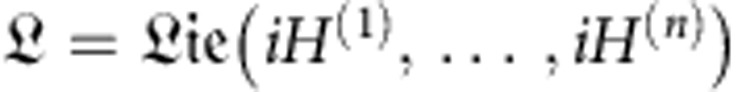 formed by the linear combinations of *iH*^(*j*)^ and their iterated commutators, 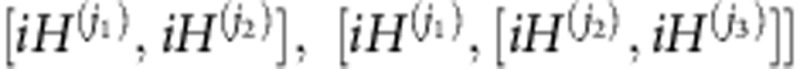 and so on. Full controllability is hence achieved if the dimension of 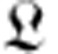 is large enough to permit the implementation of all possible unitary transformations on the system, that is, 
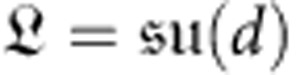
, with *d* being the dimension of the system (without loss of generality the Hamiltonians can be assumed to be traceless, since the global phase does not play any role).

Suppose now that between the applications of consecutive pulses *U*^(*j*)^, we are allowed to perform von Neumann’s projective measurements (Fig. 1b), aimed at checking whether or not the state of the system belongs to a given subspace 
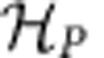
 of the global Hilbert space. Specifically, we will assume that the system is originally initialized in 
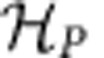
 while the various *U*^(*j*)^ are infinitesimal transformations. Under this condition, the Zeno effect can be invoked, in the limit of infinitely frequent measurements, to ensure that with high probability the system will be always found in 
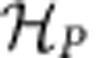
 after each measurement, following a trajectory described by the effective Hamiltonians 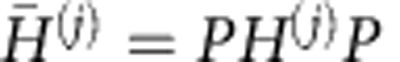, with *P* the projection onto 
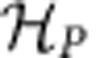
 (Fig. 1c)^8^,^11^. In other words, alternating the control pulses under frequent applications of the projection *P* the sequence 
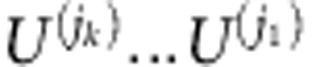
 can be effectively transformed into a rotation which on 
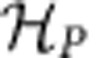
 is defined by the unitary operator 
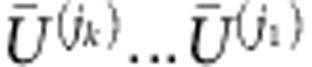
 where 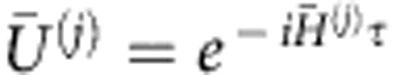. Accordingly the real Lie algebra 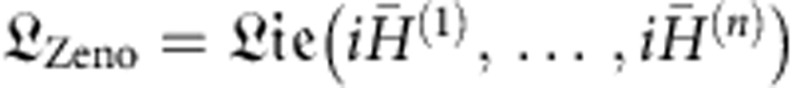 now replaces 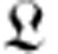 in defining the space of unitary transformations which can be forced on the system. The fundamental result of this paper is to observe that by properly choosing the system setting, the dimension of 
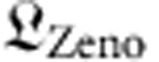
 can be made larger than 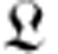, to the extent that the former can be used to fully control the system on 
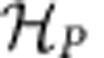
, in spite of the fact that the latter is not capable of doing the same.

**Figure 2:**
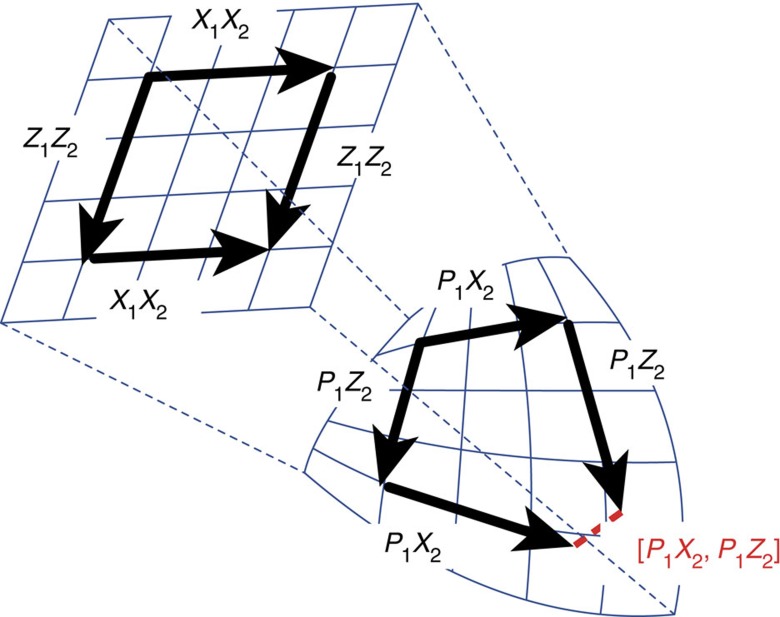
Schematics of the full versus projected system algebras. The arrows are tangents (generators) on a manifold of unitary transformations. In the larger space (upper), the operations commute, so no matter which way we go, we end up at the same point. It is not the case for the projected system (lower): the projected operations do not commute, and the gap represents the non-commutativity. Even though the projected system is embedded in a smaller space, its dynamics is more complex, because of the curvature induced by the projection: new directions can be explored.

To better elucidate the idea, we find it useful to introduce a simple example, where the system is identified with a two-qubit system with control Hamiltonians


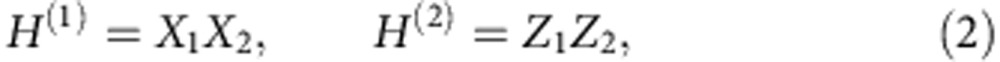


(we hereafter use *X*, *Y*, *Z* to denote Pauli operators and write tensor products as strings, specifying systems by subscripts and omitting the identity operators). Notice that their commutator vanishes [*H*^(1)^, *H*^(2)^]=0, and hence the naked algebra 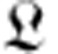 of the two-qubit system has dimension only 2. Consider now the Zeno algebra induced by the projection


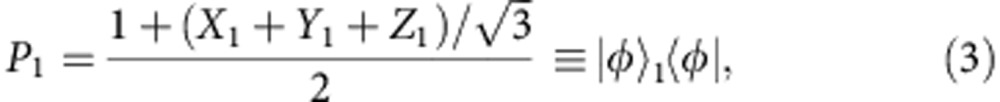


which freezes the first qubit in the state |*φ*›_1_ in the Zeno limit. Then, the effective Zeno Hamiltonians


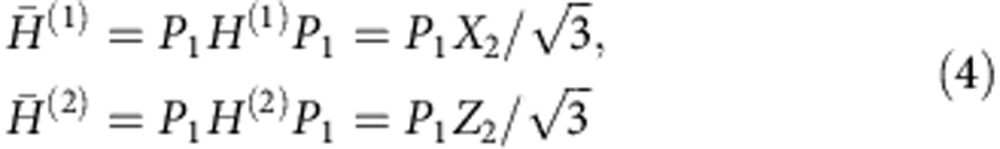


exhibit a non-trivial commutator 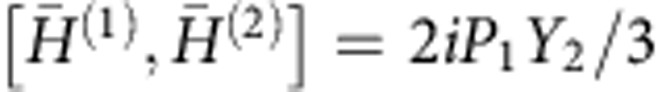, which makes the dimension of 
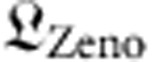
 equal to 3 (the situation is schematically illustrated in Fig. 2). This in particular implies that 
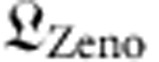
 can now be used to fully control the system in the subspace 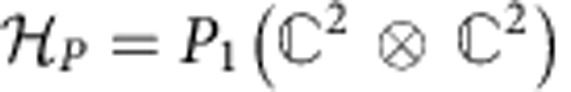 (which is isomorphic to the Hilbert space of qubit 2), a task that could not be fulfilled with the original 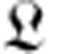.

### Zeno yields full control

The example presented in the previous paragraph clarifies that the constrained dynamics can be more complex than the original unconstrained one. The natural question arises: how big can such a difference become? To what extent can the presence of a measurement process increase the complexity of dynamics in quantum mechanics? In the following, we provide a couple of examples in which the enhancement in complexity is exponential. While the unprojected dynamics are only two or three dimensional, the projected ones are universal for quantum computation. This shows that the simple ingredient of projective measurement can strongly influence the complexity of dynamics.

**Figure 3:**
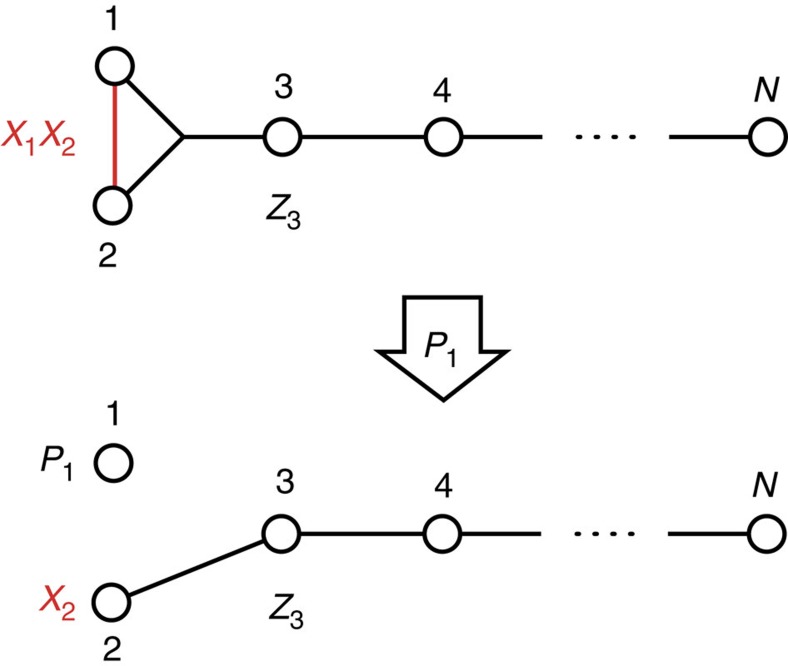
Schematics of the *N*-qubit model described in Example A. Straight edges represent the Heisenberg interactions, while the triple edge represents the three-body interaction among qubits 1–3. The red part in the upper figure corresponds to *H*^(1)^ acting on qubits 1 and 2, while the remainder including a local term *Z*_3_ on qubit 3 corresponds to *H*^(2)^ acting on all the *N* qubits. The Zeno projection *P*_1_ on qubit 1 transforms the upper Hamiltonians to the lower model, where the state of qubit 1 is frozen, while we are left with a Heisenberg chain with the local term *Z*_3_ and a control 
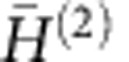
 on qubit 2. The Lie algebra of the upper system is only two dimensional, while the lower allows us to perform full control over the system apart from the frozen qubit 1.

In Example A, we consider *N* qubits (Fig. 3, upper), the first two of which are manipulated via the control Hamiltonians *H*^(1)^=*X*_1_*X*_2_, and complement it with *H*^(2)^ consisting of the nearest-neighbor Heisenberg interactions 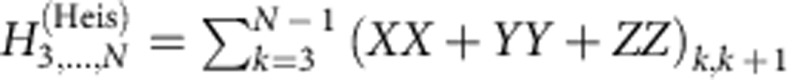 involving all the qubits but the first two, together with a coupling term acting on the first three qubits and a local term on the third, that is,


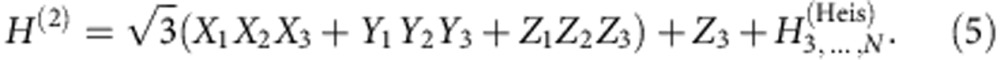


Due to the anticommutation of the Pauli operators, one can easily verify that the two Hamiltonians *H*^(1)^ and *H*^(2)^ commute with each other [*H*^(1)^, *H*^(2)^]=0, defining hence a Lie algebra 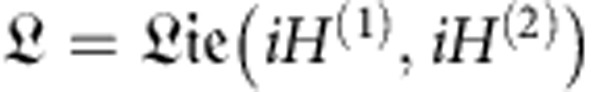, which is barely two dimensional. Now let us consider their constrained versions using the same projection *P*_1_ as in equation (3). With this choice, we have 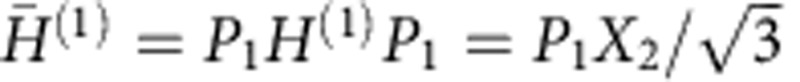, and the Zeno Hamiltonian associated to *H*^(2)^ is given by


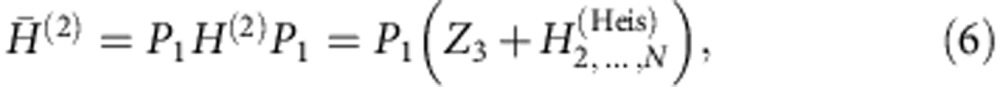


where now 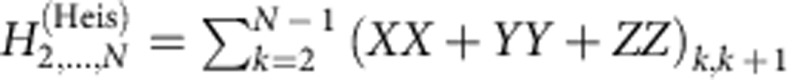 is the nearest-neighbor Heisenberg Hamiltonian acting on qubits 2,…, *N*. While qubit 1 is kept frozen in the state |*φ*›_1_ by the repetitive projections *P*_1_, the remaining *N*−1 qubits now form a Heisenberg chain with a local term on qubit 3 (Fig. 3, lower). Elementary but cumbersome calculation shows that with these Zeno Hamiltonians qubit 2 is fully controllable, which by ref. 28 implies that the whole system apart from the frozen qubit 1 is fully controllable. Consequently, we have 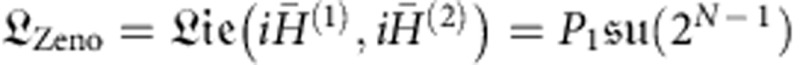, so that the Zeno algebra is of exponential size, as claimed.

The next example, Example B, is an alternative that does not involve three-body interactions. Consider, for instance, three Hamiltonians *H*^(1)^=*Z*_1_*Z*_2_, *H*^(2)^=*X*_3_*X*_4_, and 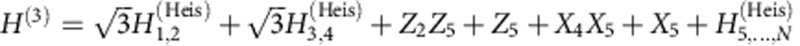, and take the Zeno projection to be *P*=*P*_1_*P*_3_ with *P*_1_ and *P*_3_ projecting qubits 1 and 3, respectively into the states |*φ*›_1_ and |*φ*›_3_ defined as in equation (3). These Hamiltonians commute with each other, and their Lie algebra 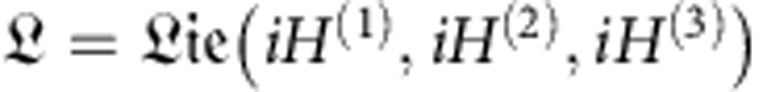 is only three dimensional. Analogously to the previous example, by exploiting the results of ref. 28, one can easily show that the dimension of 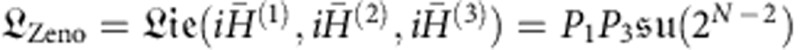 is again exponential, allowing the full control of all the qubits but the first and the third.

### Generality and Hamiltonian purification

What we have observed above is not a contrived phenomenon, but actually quite a general one. Considering the pair of Hamiltonians *H*^(1)^ and *H*^(2)^ with the projection *P*_1_ from Example A above, we can be certain that there exists a projection and a pair of commutative Hamiltonians such that the projected dynamics is essentially 
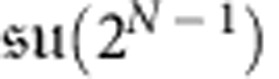
. A standard argument in control theory is that if a system is fully controllable for a specific choice of parameters, then it is also fully controllable for almost all parameters^27^. In our case, it implies that almost all commuting Hamiltonians will become universal through the Zeno projection on a single qubit (see Methods for more details).

Furthermore, we can show the converse: any non-commutative dynamics can be thought of as the projected version of commutative dynamics in a larger space. This general phenomenon is in accord with the philosophy of geometrization discussed in the introduction. In analogy with the purification of states in quantum information theory^5^, we call it Hamiltonian purification. While we give a detailed mathematical analysis elsewhere, let us present the simplest case. Consider two arbitrary *d*-dimensional Hamiltonians *h*^(1)^ and *h*^(2)^. We extend the Hilbert space by a single qubit and define their ‘purifications’ by


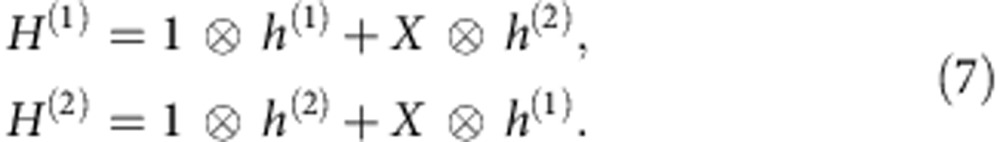


These extended Hamiltonians *H*^(1)^ and *H*^(2)^ are easily seen to commute with each other, [*H*^(1)^, *H*^(2)^]=0, and the projection by *P*=(1+*Z*)⊗1/2 yields 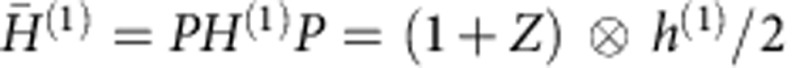 and 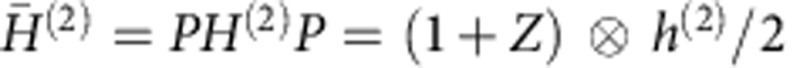, which act as *h*^(1)^ and *h*^(2)^ in the original space before the extension. We can furthermore apply this procedure iteratively to larger sets of Hamiltonians, which means that any complex dynamics can be thought of as a simple one taking place on a larger space, with the complexity arising only from projections.

### Local noise yields full control

In a classical setting, the measurement process is typically perceived as a passive resource that enforces control only when properly inserted in a feedback loop. As explicitly shown by our analysis, and more generally by the results of refs 9, 10, 11, 12, 13, 14, 15, 16, 17, 18, 19, 20, 21, 22, this is no longer the case in quantum mechanics: measurements can indeed be used to directly drive a quantum system even in the absence of a feedback mechanism.

Interestingly enough, for the control scheme we are analysing here, measurement is not the only way to implement the required projection *P*. The same effect is attainable by fast unitary kicks and by strong continuous coupling^8^,^11^,^29^. Furthermore, owing to the non-adaptive character of the procedure (we never need to use the measurement outcomes to implement the control), it is also achievable by tailoring a strong dissipative process^21^,^30^,^31^,^32^. The latter option is of particular interest for us since, along the line of refs 33, 34, 35, it points out the possibility of taking advantages of the interaction of the system of interest with an external environment, which is typically considered detrimental for quantum processing.

Specifically, for the qubit chain analysed above (Example A), one can show that the action of a simple amplitude damping channel^5^ can raise the dynamical complexity to the level of universal quantum computation. In fact, the decay process bringing qubit 1 to the state |*φ*›_1_ can act as a projection *P*_1_ (see Methods), and in the strong-damping limit it is effective in inducing a quantum Zeno effect on qubit 1, yielding the full Lie algebra 
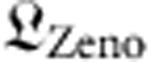
 in the rest of the qubit chain. Moreover, due to the same reasoning as the one outlined above, almost all qubit amplitude damping channels induce exponential complexity.

A comment regarding the presence of further decoherence, besides the one employed to enforce the Zeno limit, is in order. It is clear that additional decoherence will be detrimental for the performance of the scheme. Accordingly, one should adopt computational schemes that protect the encoded information from the action of this noise (for example, exploiting quantum error correction embeddings). In a sense, the view is to identify two kinds of noise sources: the ‘good’ noise (which can be exploited to induce control via the Zeno effect) and the ‘bad’ noise, which instead has to be controlled and removed. Whether or not a given system will allow such separation cannot be decided *a priori*: to answer this question, one needs to look at the specific properties of the system under consideration.

Similarly, the problems arising from the finiteness of the measurement procedure (or the application of wrong projections) are complex and model dependent. In the approach we propose, the environment performs a kind of ‘adaptive’ feedback. The scheme, in its generality, appears to be feasible and robust. Clearly, errors will inevitably lead to quantitative deterioration, but we expect that the main positive qualitative features will still be present when additional factors will be taken into account. These are all topics of practical interest, to be addressed in the future.

## Discussion

The schemes presented in this work are not meant to implement an efficient quantum computer themselves (note, however, ref. 36), but rather to provide a novel method for control, which might find applications in quantum information processing. Instead they should be viewed as a proof of the fact that generally adding a simple projection or noise to a dynamical system can profoundly modify the global picture and provoke a drastic increase in complexity. This bears some similarities to measurement-based quantum computation^37^,^38^ and related schemes^39^,^40^, although there are important differences, in that one does not require the system to be initialized in a complex state; the measurement is constant; and its outcome is not used adaptively in future computations^41^.

Our results can be presented as a quantum version of the Plato’s Cave allegory^42^. In the original version of the myth, the reality perceived within the Cave is described by the projected shadows of some more fundamental dynamics (‘the Ideals’), which is intrinsically more simple (‘intelligible’). In the quantum world, the projection plays a more active role, making the dynamics of the associated quantum shadows as complex as universal quantum computation and, conversely through Hamiltonian purification, making non-commutative dynamics simple.

## Methods

### Sketch of the proof of the generality

We found the two commuting Hamiltonians *H*^(1)^ and *H*^(2)^ in the *N*-qubit model depicted in Fig. 3 (Example A), whose projected counterparts 
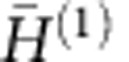
 and 
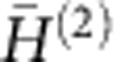
 with the projection in equation (3) of the structure *P*=*P*_1_⊗1 generate 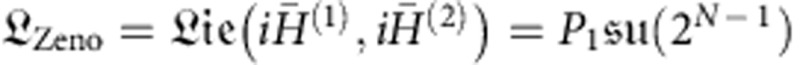. This single example makes us sure that it is the case for almost all systems.

To see this, let us formalize in the following way. Take (*H*^(1)^, *H*^(2)^, *P*) of Example A again. We extract the relevant sector specified by *P* from each element of 
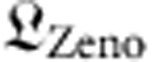
 and call it *L*_*j*_ (*j*=1,…,*d*^2^−1), which is a *d* × *d* matrix with dimension *d*=2^*N*−1^ and is a function *L*_*j*_=*L*_*j*_(*H*^(1)^, *H*^(2)^) of *H*^(1)^ and *H*^(2)^. Together with the *d* × *d* identity matrix *L*_0_=1, the matrices {*L*_*j*_} form 
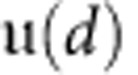
. This fact can be mathematically expressed as follows. We ‘vectorize’ each matrix *L*_*j*_ to a *d*^2^-dimensional column vector |*L*_*j*_) by lining up the columns of the matrix *L*_*j*_ from top to bottom, and gather the column vectors |*L*_*j*_) side by side to make up a *d*^2^ × *d*^2^ matrix *L*=(|*L*_0_)…|*L*_*d*^2^−1_)). Then, the fact that the matrices {*L*_*j*_} span 
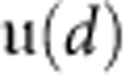
 is expressed as *D*=det *L*≠0. Note that this determinant is also a function *D*=*D*(*H*^(1)^, *H*^(2)^) of *H*^(1)^ and *H*^(2)^.

Now take a generic pair of commuting Hamiltonians 
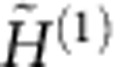
 and 
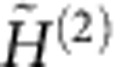
 of *N* qubits, that is, we randomly choose their eigenvalues 
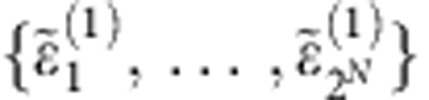
, 
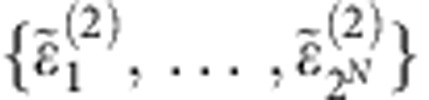
 and a common unitary matrix 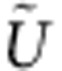 that diagonalizes 
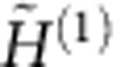
 and 
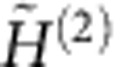
 simultaneously. Inserting this pair of Hamiltonians, the determinant 
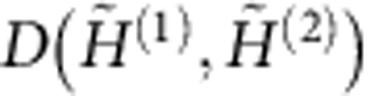
 is, by construction, a polynomial in the parameters 
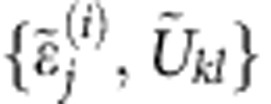
 (*i*=1, 2; *j*, *k*, *l*=1,…, 2^*N*^). We already know that this polynomial is non-vanishing for the parameter set 
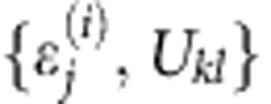
 corresponding to the above specific choice of the Hamiltonians *H*^(1)^ and *H*^(2)^. Therefore, the determinant *D* is a non-zero polynomial in the parameters 
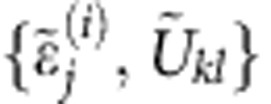
, implying that its roots are of measure zero in the parameter space. In other words, for almost all parameters 
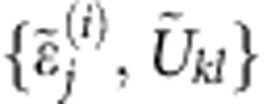
, the determinant *D* is non-vanishing, and in turn, almost all pairs of commuting Hamiltonians become universal, generating 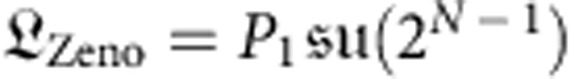, by the projection *P* on the first qubit. This argument can be generalized to any rank 2^*N*−1^ projection, and also to any qubit amplitude damping channel in the strong-damping limit.

### Projection by amplitude damping channel

The continuous projection *P*_1_ required for the qubit-chain model depicted in Fig. 3 can be induced by an amplitude damping channel acting on qubit 1. In fact, consider the master equation 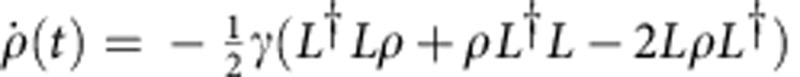 with a single Lindblad operator *L*=|*φ*›_1_‹*φ*_⊥_|, which describes the decay of qubit 1 from |*φ*_⊥_›_1_ to |*φ*›_1_, where |*φ*›_1_ is associated with the projection *P*_1_ in equation (3) and |*φ*_⊥_›_1_ is the state orthogonal to |*φ*›_1_. Solving the system dynamics under the master equation yields *ρ*(*t*)=(1−*e*^−*γt*^) × *P*_1_Tr_1_*ρ*(0)+*e*^−*γt*^[*P*_1_*ρ*(0)*P*_1_+*Q*_1_*ρ*(0)*Q*_1_]+*e*^−*γt*/2^[*P*_1_*ρ*(0)*Q*_1_+*Q*_1_*ρ*(0)*P*_1_], where *Q*_1_=1−*P*_1_ and Tr_1_ represents the partial trace over qubit 1. Thus, in the limit *γt*→∞, we have *ρ*(*t*)→*P*_1_Tr_1_*ρ*(0), and qubit 1 is projected into the state |*φ*›_1_ with probability 1. If this process takes place on a timescale *γ*^−1^ much shorter than any other timescales involved in the dynamics or the controls, then it is effective in inducing a quantum Zeno effect on qubit 1, and it is essentially equivalent to repeating projective measurements.

## Author contributions

All authors have contributed equally to this paper.

## Additional information

**How to cite this article**: Burgarth, D. K. *et al.* Exponential rise of dynamical complexity in quantum computing through projections. *Nat. Commun.* 5:5173 doi: 10.1038/ncomms6173 (2014).
